# Measurement equivalence and feasibility of the EORTC QLQ-PR25: paper-and-pencil versus touch-screen administration

**DOI:** 10.1186/1477-7525-12-23

**Published:** 2014-02-20

**Authors:** Yu-Jun Chang, Chih-Hung Chang, Chiao-Ling Peng, Hsi-Chin Wu, Hsueh-Chun Lin, Jong-Yi Wang, Tsai-Chung Li, Yi-Chun Yeh, Wen-Miin Liang

**Affiliations:** 1 Graduate Institute of Public Health, China Medical University, Taichung, Taiwan; 2 Epidemiology and Biostatistics Center, Changhua Christian Hospital, Changhua, Taiwan; 3 Graduate Institute of Biostatistics, China Medical University, No.91 Hsueh-Shih Road, Taichung 40402, Taiwan; 4 Buehler Center on Aging, Health & Society, Northwestern University Feinberg School of Medicine, Chicago, IL, USA; 5 Department of Urology, China Medical University Hospital, Taichung, Taiwan; 6 School of Medicine, China Medical University, Taichung, Taiwan; 7 Department of Health Risk Management, China Medical University, Taichung, Taiwan; 8 Department of Health Services Administration, China Medical University, Taichung, Taiwan; 9 Biostatistics Center, China Medical University, Taichung, Taiwan

**Keywords:** Health-related quality of life, Prostate cancer, EORTC QLQ-PR25, Equivalence, Feasibility, Cross-over design, Touch-screen, Paper-and-pencil

## Abstract

**Objective:**

We assessed the measurement equivalence and feasibility of the paper-and-pencil and touch-screen modes of administration of the Taiwan Chinese version of the EORTC QLQ-PR25, a commonly used questionnaire to evaluate the health-related quality of life (HRQOL) in patients with prostate cancer in Taiwan.

**Methods:**

A cross-over design study was conducted in 99 prostate cancer patients at an urology outpatient clinic. Descriptive exact and global agreement percentages, intraclass correlation, and equivalence test based on minimal clinically important difference (MCID) approach were used to examine the equity of HRQOL scores between these two modes of administration. We also evaluated the feasibility of computerized assessment based on patients’ acceptability and preference. Additionally, we used Rasch rating scale model to assess differential item functioning (DIF) between the two modes of administration.

**Results:**

The percentages of global agreement in all domains were greater than 85% in the EORTC QLQ-PR25. All results from equivalence tests were significant, except for Sexual functioning, indicating good equivalence. Only one item exhibited DIF between the two modes. Although nearly 80% of the study patients had no prior computer-use experience, the overall proportion of acceptance and preference for the touch-screen mode were quite high and there was no significant difference across age groups or between computer-use experience groups.

**Conclusions:**

The study results showed that the data obtained from the modes of administration were equivalent. The touch-screen mode of administration can be a feasible and suitable alternative to the paper-and-pencil mode for assessment of patient-reported outcomes in patients with prostate cancer.

## Introduction

The proper use of patient-reported outcome (PRO) measurement in clinical settings has become increasingly important to obtain more comprehensive information to guide clinical decision-making, treatment planning, and clinical management [1]. Traditionally, PRO data are collected through face-to-face interviews or patient self-report to paper-based questionnaires, which is labor intensive and time consuming. With the emergence of computer technology, electronic methods of data collection (e.g., touch-screen response or interactive voice response) are becoming more popular and viable alternatives to conventional surveys carried out in clinical practice [1-3].

Electronically administered questionnaires allow data to be automatically entered real time into a database, after which the score is immediately calculated; thus, data coding errors and the workload of health professionals are reduced [3,4]. The time required by the patient to complete the electronically administered questionnaire such as electronic patient-reported outcome (ePRO) questionnaire, is also reduced for routine clinical practice [3,5]. In addition, increased use of the ePRO questionnaires in clinical assessments may promote integration of PRO and clinical information. Once patients have completed the ePRO questionnaires during their clinic visits, their item responses will be automatically scored and summarized for potential clinical use with other patient-related information. The integrated results are readily available in easily interpretable reports that can be viewed together by the clinician and their patient during clinical encounter. Therefore, the process can enhance the efficiency and quality of healthcare and patient-physician communications [3,6,7]. Nonetheless, the equivalence of the ePRO version and its original paper-and-pencil version should be thoroughly evaluated, and the patient preference and acceptance should also be examined before shifting from paper-and-pencil data to ePROs without demonstrating its feasibility [6,8]. Some studies have examined and validated the measurement equivalence of paper-and-pencil-based version and touch-screen computer-based version; the results showed that the data collected from paper- and computer-administered PROs were very similar and the touch-screen version was well accepted by most subjects [6-10].

Prostate cancer is a common disease among men in many Western countries and developed Asian countries. The standardized incidence in Taiwan (adjusted by the 2000 world population) has increased from 1.86 per 100,000 men in 1979 to 28.77 per 100,000 men in 2010 [11]. Moreover, long-term survival results have shown that health-related quality of life (HRQOL) has become an important outcome measure in different clinical settings [12]. The European Organization for Research and Treatment of Cancer (EORTC) Quality of Life Study Group developed the EORTC QLQ-PR25, a 25-item questionnaire designed for use among patients with localized and metastatic prostate cancer, is a commonly used tool to assess HRQOL in patients with prostate cancer. It includes four domains that assess urinary symptoms, bowel symptoms, treatment-related symptoms, and sexual activity and functioning. The results of international field validation have been published in 2008 [13]. The paper-and-pencil versions of the EORTC QLQ-PR25 have satisfactory reliability and validity [12-14]. In this study, we used the Taiwan Chinese version of the EORTC QLQ-PR25 questionnaire published by Chie et al. in 2010 [12]. It was also shown to be reliable and valid to assess HRQOL using the modern test theory approach in our previous study [15]. To the best of our knowledge, the psychometric properties and feasibility of the touch-screen version of this questionnaire for prostate cancer patients have not been well established, and no data have been reported in Taiwan. Therefore, in this study we sought to assess the measurement equivalence and feasibility of the paper-and-pencil and touch-screen versions of the EORTC QLQ-PR25 in patients with prostate cancer in Taiwan.

## Methods

### Research design and data collection

A randomized cross-over design was used in this study. A total of 107 prostate cancer patients in various stages of illness and treatment were enrolled from September 2008 to October 2009 in the Department of Urology outpatient clinic of China Medical University Hospital in Central Taiwan. Patients who could not read, speak, or write Chinese were excluded. All patients provided written informed consent. The study procedures were approved by the Institutional Review Board of the China Medical University Hospital.

**Figure 1:**
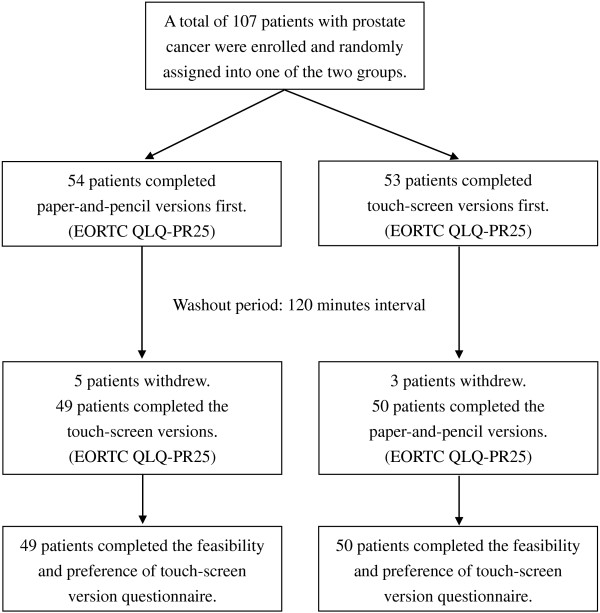
Study structure in the study.

Initially, 107 patients with prostate cancer were enrolled and randomly assigned into one of the two study groups, with 54 patients in the paper-and-pencil-version-first group (denoted as paper/touch-screen group) and 53 patients in the touch-screen-version-first group (denoted as touch-screen/paper group). Each patient was asked to complete both versions of the questionnaires. Patients in the paper/touch-screen group were given the paper-and-pencil version of the questionnaire first, followed by the touch-screen version after a 120-minute interval. By contrast, the touch-screen/paper patients were given the touch-screen questionnaire first, followed by the paper-and-pencil version 120 minutes later. Once the patients finished the first mode of administration, they were led to the education room to watch health education videos. This was to dilute the memory of their responses to the questions from the first administration as the same set of question were asked at the second administration. After completing each questionnaire for the two modes of administration, each patient was asked to answer a usability and feasibility questionnaire to indicate their preference and acceptance of the touch-screen version of the questionnaire. Ninety-nine patients successfully completed both assessments and eight patients had to leave early and did not complete the whole procedure. The study scheme is shown in Figure 1.

### HRQOL measures

The prostate-specific module EORTC QLQ-PR25 is a self-administered questionnaire that includes 4 subscales for assessment of Urinary symptoms (9 items, labeled US31–US39), Bowel symptoms (4 items, BS40–BS43), Hormonal treatment-related symptoms (6 items, TS44–TS49), and Sexual activity and function (6 items, SX50–SX55). In this study, no patients reported using incontinence aid, therefore, the item (US38, “Has wearing an incontinence aid been a problem for you? Answer this question only if you wear an incontinence aid.”) was excluded from the Urinary symptom domain for analysis. Each of the 24 retained items was scored from 1 to 4 (1 = “Not at all”, 2 = “A little”, 3 = “Quite a bit”, and 4 = “Very much”) [13]. Domain scores of the EORTC QLQ-PR25 were linearly transformed to a 0 to 100 scale based on its scoring manual, which requires answering at least half of the total number of items in the domain. Higher scores reflected either more symptoms (e.g., Urinary, Bowel, and Hormonal treatment-related symptoms) or higher levels of functioning (e.g., Sexual activity and function) [16].

### Setting of the touch-screen version

The touch-screen version was developed by a team of physicians specializing in prostate cancer, technicians with expertise in system design and programming, epidemiologists, and statisticians. Java software was used to develop the system using an Oracle database [17].

### Statistical analysis

Descriptive statistics for continuous variables are presented as means and standard deviations, whereas categorical variables are presented as frequencies and proportions. We assessed the differences in demographic characteristics and time to complete the questionnaire between the two study groups using independent *t*-test for continuous data and Chi-square or Fisher’s exact test for categorical data. And a paired *t*-test was used to compare the administration time between two modes.

We used a mixed model to assess the quality of the cross-over design, wherein the dependent variable was the domain score and the main independent variables were administration order (order effect), mode type (mode effect), and their interaction (carry-over effect). A random effect accounted for the covariance structure induced by the repeated measures. Gender and age effects were also included for adjustment of confounding effect. After testing the mode-by-order interaction, we fit the model again without interaction term (i.e., main-effect model) if the carry-over effect is not statistically significant at 5% level. No significant carry-over effect in the interaction model and no significant order effect in the main-effect model indicated methodologically appropriate for the cross-over design.

The agreement of scores between the paper and the computerized administrations was assessed at the individual patient level. “Exact agreement” referred to as patients who provided the same responses to individual questions on both paper-and-pencil- and touch-screen-administered questionnaires. “Global agreement” was defined as the proportion of agreement within one adjacent response category in either higher or lower direction [18]. We also used intraclass correlations (ICC) calculated from a random-effects mixed model and interpreted the results based on criteria proposed by Bartko et al. [19] and Stokdijk et al. [20] as follows: an ICC < 0.40 indicated poor agreement, 0.40 to 0.59 indicated moderate agreement, 0.60 to 0.75 indicated good agreement, and > 0.75 indicated excellent agreement. Highly positive ICCs indicate that paper and computer measures covary, and the mean and variability of the scores are similar [2].

In addition, we used a “Two One-Sided Test” procedure to determine whether the two administrations produce equivalent results. Equivalence testing is operationally different from the conventional method (e.g., independent *t*-test), which is mainly used to detect difference rather than equivalence. Equivalence testing refers to a trial wherein the primary objective is to show that the response to the novel intervention is as good as the response to the standard intervention. This procedure begins with attempting to demonstrate that they are equivalent within a practical, preset limit *δ* (i.e., |*μ*_*paper*_ − *μ*_*touch* − *screen*_| < *δ*), and sets a null hypothesis that the two mean values are not equivalent (i.e., |*μ*_*paper*_ − *μ*_*touch* − *screen*_| ≥ *δ*). The method we used is computationally identical to perform two one-sided *t*-tests with the following sets of hypotheses:

$$\begin{array}{l} \;\mathrm{Left}\;\mathrm{tail}\;\mathrm{Right}\;\mathrm{tail}\\ \left\{\begin{array}{l} H_{0}:D=\mu_{\mathrm{paper}}-\mu_{\mathrm{touch}-\mathrm{screen}}\leq ‒\delta \\ H_{1}:D=\mu_{\mathrm{paper}}-\mu_{\mathrm{touch}-\mathrm{screen}}>‒\delta \end{array}\right)\;\left\{\begin{array}{l} H_{0}:D=\mu_{\mathrm{paper}}-\mu_{\mathrm{touch}-\mathrm{screen}}\geq \delta \\ H_{1}:D=\mu_{\mathrm{paper}}-\mu_{\mathrm{touch}-\mathrm{screen}}<\delta \end{array}\right) \end{array}$$

This premise is conceptually opposite to that of the conventional independent *t*-test procedure [21]. The occurrence of both rejections from two one-sided *t*-tests at 5% significant level indicates that the two modes are equivalent, which means that the difference between the groups is not more than a tolerably small amount (i.e., |*μ*_*paper*_ − *μ*_*touch* − *screen*_| < *δ*) [22,23]. This small amount of allowable difference is the margin that defines the “zone of indifference” where the interventions are considered equivalent [24]. In our analysis, we used a minimum clinically important difference (MCID) of 5 (i.e., *δ* = 5) based on previous studies [25,26], as the tolerable amount to assess equivalence.

The required sample size for this study was based on the assumption that no clinical differences are present between the domain scores of the two administration modes under a cross-over design study. The MCID for each domain score of the EORTC QLQ-PR25 was set at a five-point score, and the standard error of the domain score was set at 8 based on the empirical data. The minimum sample size was estimated to be 80 using the statistical software PASS to detect an equivalence difference of 5 with 80% power and 5% type I error.

### Confirmation from modern measurement theory

Rasch analysis, based on the modern measurement theory, has been shown to be a useful tool for the development of new PRO measures and in evaluating the measurement structure of existing PRO measures [27,28]. We used the rating scale model (RSM) to estimate difficulty calibration for each item. RSM, an extension of the dichotomous Rasch model, for polytomous items with ordered response categories was chosen as it is suitable for the items used in this study. In Rasch analysis, differential item functioning (DIF) can be used to examine item measurement invariance [29,30]. In this study, DIF, an item lacking equivalence in performance across the two groups or settings (e.g., paper-and-pencil vs. touch-screen administration), was identified statistically by conducting an independent *t*-test on the difficulty calibration of each item. An item was said to exhibit DIF if the test was significant (*p* < 0.05) [31].

## Results

### Demographic characteristics

Table 1 shows the demographic characteristics of the study patients with prostate cancer. The mean age was 70.1 years in the paper-and-pencil/touch-screen group and 69 years in the touch-screen/paper-and-pencil group. The age range of patients was 57 years to 87 years. More than half of the participants graduated from high school. Approximately 80% of the patients had no experience using a computer. No statistically significant differences were observed for demographic characteristics between the two groups.

**Table 1:** Demographic characteristics and time for completion of questionnaires of the two groups of prostate cancer patients

	**Paper/Touch-screen**	**Touch-screen/Paper**	
	**(n = 49)**	**(n = 50)**	***p*-value**
Age (year) (N (%))			
<= 65	12 (24.5)	13 (26.0)	0.778^b^
66-70	11 (22.5)	14 (28.0)	
71-75	12 (24.5)	13 (26.0)	
> 75	14 (28.5)	10 (20.0)	
Mean (SD)	70.1 (7.6)	69.0 (8.1)	0.463^a^
Education level (N (%))			0.701^b^
College or above	14 (29.8)	13 (26.0)	
Senior high	11 (23.3)	15 (30.0)	
Junior high	11 (21.4)	7 (14.0)	
Primary school or less	12 (25.5)	15 (30.0)	
Previous experience using computers (N (%))			
Yes	10 (20.4)	9 (18.0)	0.837^b^
No	39 (79.6)	41 (82.0)	
Time for completion of questionnaires (min)^d^			
Paper version (Mean (Range))	17.9 (5.0 ~ 39.0)	14.7 (6.0 ~ 31.0)	0.022^a^
Touch-screen version (Mean (Range))	15.7 (5.0 ~ 30.0)	20.5 (9.0 ~ 41.0)	0.002^a^
*p*-value	0.516^c^	<0.001^c^	

^a^*p*-value was evaluated by Independent *t*-test.

^b^*p*-value was evaluated by Chi-square test.

^c^*p*-value was evaluated by Paired *t*-test.

^d^Time for completion of the two questionnaire modes by four questionnaires, including the EORTC QLQ-C30, EORTC QLQ-PR25, IIEF-5 (International Index of Erectile Function), and IPSS (International Prostate Symptom Score).

### Mixed model analysis

We conducted the mixed model with two main effects (accounted for the mode effect and the order effect) and their interaction (accounted for the carry-over effect). No carry-over effects were found (*p*-value > 0.05). We then removed the interaction and reran the model. No order effect was observed (*p*-value > 0.05). The results confirmed the quality of the cross-over design.

### Exact and global agreement analysis

Table 2 shows the percentages of exact and global agreements for each domain. In the “urinary symptoms” domain that included 8 items, 791 paired responses (8 items × 99 subjects – 1 missing pair) were noted. Out of 791, 629 paired responses were identical, which yielded an exact agreement percentage of 80% (= 629/791). Our results showed that the percentages of exact agreement for all domains in EORTC QLQ-PR25 ranged from 61% to 89%. The percentages of global agreement (i.e., the difference of scores of each paired responses was within one response category) ranged from 88% to 100%.

**Table 2:** Exact and global agreements and ICC between touch-screen and paper-and-pencil modes

**EORTC QLQ-PR25**	**Exact agreement**^**a**^	**Global agreement**^**b**^	**ICC**
Urinary symptoms (8 items)^c^	629/791 (80%)	778/791 (98%)	0.78
Bowel symptoms (4 items)	351/396 (89%)	396/396 (100%)	0.72
Treatment-related symptoms (6 items)	500/591 (85%)	574/591 (97%)	0.47
Sexual activity (2 items)	142/196 (72%)	187/196 (95%)	0.61
Sexual functioning (4 items)^d^	92/151 (61%)	133/151 (88%)	0.45

Number of total pairs = number of subjects × number of items – number of missing pairs.

^a^Exact agreement (in%): number of same response pairs/number of total pairs.

^b^Global agreement (in%): number of within one-difference response pairs/number of total pairs.

^c^In our study, there is no patients reported using incontinence aid, therefore, we deleted an item (US38 “Incontinence aid”) from the Urinary symptom domain, resulting in 8 items remained in this domain.

^d^Sexual functioning items only apply to sexually active subjects. A total of 48 patients reported being sexually active. Meanwhile, forty-one missing pairs occurred in this domain.

### Intraclass correlation coefficient analysis

The intraclass correlation coefficients (ICCs) ranged from 0.45 (“Sexual Function” domain) to 0.78 (“Urinary symptoms” domain) for all the domains in the EORTC QLQ-PR25 (Table 2), indicating moderate to excellent agreement for each domain between two modes.

### Equivalence test based on minimal important difference approach

Table 3 shows the results of the domain scores and equivalence test based on the MCID approach for comparison of touch-screen and paper-and-pencil modes. Equivalence tests based on the MCID of 5 were used to assess the equivalent properties between the two modes and the results showed the measurement scales between two modes were equivalent for all domains except for Sexual functioning domain.

**Table 3:** Mean scores and equivalence test between touch-screen and paper-and-pencil modes

	**Paper-and-pencil**		**Touch-screen**		**Equivalence test**^**c**^		
**EORTC QLQ-PR25**^**a**^	**Mean**	**SD**	**Mean**	**SD**	**95% CI of**	**Left tail**^**e**^	**Right tail**^**e**^
					**Mean difference**^**d**^	***p*-value**	***p*-value**
Urinary symptoms (8 items)	19.5	13.5	21.1	13.3	−3.3 to 0.1	<0.001	<0.001
Bowel symptoms (4 items)	5.3	8.6	5.7	7.9	−1.5 to 1.0	<0.001	<0.001
Treatment-related symptoms (6 items)	11.1	10.2	10.3	9.2	−1.1 to 2.9	<0.001	<0.001
Sexual activity (2 items)	19.4	20.2	20.2	20.8	−5.0 to 2.3	0.048	<0.001
Sexual functioning (4 items)^b^	62.3	22.0	61.8	20.9	−5.7 to 9.5	0.076	0.415

^a^All scores were linearly converted to a 0 to 100 scale, with higher scores indicating a higher level of functioning and more severe symptoms.

^b^Sexual functioning items are conditional and only applicable to those being sexually active. A total of 48 patients reported being sexually active in this study.

^c^Based on the equivalence test using the score 5 as the tolerably difference.

^d^Mean difference = paper-and-pencil mean score - touch-screen mean score.

^e^Left tail significance indicates *μ*_paper_ – *μ*_touch-screen_ > −5. Right tail significance indicated *μ*_paper_ – *μ*_touch-screen_ < 5. Therefore, both tails significance indicates | *μ*_paper_ – *μ*_touch-screen_ | < 5.

### DIF analysis

Table 4 shows the results of DIF of Rasch analysis. No DIF was found for EORTC QLQ-PR25 for prostate cancer patients, except item 31 (“Urinary frequency during daytime”). In general, the measurement properties for each item were equivalent between the two modes.

**Table 4:** Using Rasch analysis with rating scale model in differential item functioning analysis

	**EORTC QLQ-PR25**	**Paper**		**Computer**		**Differential item functioning**		
	**Items ranked by difficulty**	**Difficulty**	**SE**	**Difficulty**	**SE**	**Difficulty**	**SE**	***p*-value**
**Urinary symptoms (US)**^**a**^								
US37	Painful voiding (least frequent)	−3.055	0.369	−2.790	0.321	−0.266	0.489	0.588
US39	Limitation of daily activities because of US	−1.035	0.250	−1.306	0.251	0.271	0.354	0.445
US36	Urinary incontinence	−0.199	0.225	−0.349	0.226	0.150	0.319	0.638
US35	Need to remain close to toilet	−0.149	0.223	−0.346	0.226	0.197	0.318	0.536
US31	Urinary frequency in daytime	0.599	0.210	1.261	0.200	−0.662	0.290	0.024
US34	Sleep deprivation because of US	1.118	0.206	0.766	0.207	0.352	0.292	0.229
US33	Urinary urgency	1.118	0.206	1.420	0.198	−0.302	0.286	0.292
US32	Nocturia (most frequent)	1.537	0.204	1.301	0.199	0.236	0.285	0.410
**Bowel symptoms (BS)**^**a**^								
BS42	Fecal blood (least frequent)	−0.656	0.413	−0.839	0.404	0.183	0.578	0.752
BS41	Fecal incontinence	−0.656	0.413	−0.535	0.377	−0.120	0.559	0.830
BS40	Limitation of daily activities because of BS	0.437	0.339	0.313	0.328	0.124	0.472	0.793
BS43	Bloated feeling (most frequent)	0.879	0.327	1.026	0.315	−0.147	0.454	0.747
**Treatment-related symptoms (TS)**^**a**^								
TS45	Breast tenderness (least frequent)	−1.676	0.384	−1.489	0.381	−0.187	0.541	0.731
TS46	Swelling in legs or ankles	−0.449	0.257	−0.151	0.247	−0.298	0.357	0.406
TS44	Hot flushes	−0.384	0.253	−0.812	0.301	0.429	0.393	0.277
TS47	Bother due to weight loss	−0.035	0.234	0.284	0.221	−0.320	0.322	0.322
TS48	Bother due to weight gain	0.668	0.200	0.515	0.209	0.153	0.289	0.598
TS49	Felt less masculine (most frequent)	1.756	0.165	1.677	0.167	0.078	0.235	0.739
**Sexual activity (SX)**^**b**^								
SX50	Sexual interest (more likely)	−0.977	0.334	−1.338	0.315	0.362	0.459	0.432
SX51	Sexual activity (less likely)	0.968	0.344	1.359	0.330	−0.391	0.477	0.414
**Sexual functioning (SX)**^**b,c**^								
SX55	Sexual comfort (more likely)	−1.945	0.303	−1.408	0.270	−0.537	0.406	0.189
SX54	No ejaculation problems	−0.607	0.228	−0.257	0.220	−0.350	0.316	0.272
SX53	No erectile problems	−0.047	0.210	−0.047	0.214	0.000	0.299	1.000
SX52	Sexual enjoyment (less likely)	2.441	0.220	1.888	0.192	0.552	0.292	0.061

^a^Raw responses: 1: Very much; 2: Quite a bit; 3: A little; 4: Not at all, higher score means less frequent symptom.

^b^Raw responses: 4: Very much; 3: Quite a bit; 2: A little; 1: Not at all, higher score means more activity/functioning.

^c^Sexual functioning items are conditional and only applicable to those being sexually active. A total of 48 patients reported being sexually active in this study.

Difficulty: Item difficulty, higher difficulty means worse HRQOL/more frequent symptom.

### Acceptance and preference for touch-screen mode

Table 5 shows the patients’ views about the use of touch-screen questionnaire administration. Approximately 92% of patients reported that the touch-screen questionnaire administration was easy to use and approximately 97% thought the user interface was friendly. Approximately 92% of patients stated that they liked using the touch-screen to complete the questionnaire. More than two-thirds (67%) of the patients said they preferred using the touch-screen mode to fill out the questionnaire, while 30% preferred using the paper-and-pencil mode. Based on the results stratified by age, higher percentages of acceptance and preference were observed in patients < = 70 years of age compared with patients aged > 70 years. However, no statistically significant difference for each question was observed when the two age groups were compared. One hundred percent of prostate cancer patients with experience using a computer and 90% to 96% of patients without previous computer experience believed that the touch-screen mode was user friendly. Although nearly 80% of the prostate cancer patients had no computer experience, the overall percentages of acceptance and preference for the touch-screen mode was quite high and was non-significantly different compared with the results between the patients with and without computer experience.

**Table 5:** Feasibility and preference assessments for the touch-screen mode

		**Age group**			**Computer experience**		
	**All**	**<= 70**	**> 70**	***p*-value**	**Yes**	**No**	***p*-value**
	**(n = 99)**	**(n = 50)**	**(n = 49)**		**(n = 19)**	**(n = 80)**	
Feeling touch-screen mode was easy to use.	91.9%	96.0%	87.0%	0.159^a^	100.0%	90.0%	0.347^a^
Feeling user-interface of touch-screen mode was user-friendly.	97.0%	100.0%	93.9%	0.117^a^	100.0%	96.3%	0.999^a^
Expressing they liked touch-screen mode.	91.9%	96.0%	87.8%	0.159^a^	100.0%	90.0%	0.347^a^
Which version did you like?							
Neither	2.0%	0.0%	4.1%	0.117^b^	5.3%	1.3%	0.639^b^
Paper and pencil mode	30.3%	24.0%	36.7%		31.6%	30.0%	
Touch-screen mode	66.7%	74.0%	59.2%		63.2%	67.5%	
Both	1.0%	2.0%	0.0%		0.0%	1.3%	

^a^*p*-value was evaluated by Fisher’s exact test.

^b^*p*-value was evaluated by Chi-square test to compare two groups: touch-screen mode and the others (three versions combined).

## Discussion

The measurement equivalence between paper-based versions and touch-screen versions of the questionnaires has been previously demonstrated in various diseases [4,7,18,32], but to the best of our knowledge no data on the EORTC QLQ-PR25 in Taiwan have been reported. Our results showed the percentages of global agreement in all EORTC QLQ-PR25 domains were > 85%. All results from equivalence tests were significant, indicating measurement equivalence, except for Sexual functioning domain. The results of measurement equivalence were confirmed using the modern test theory approach. Only one out of 24 items exhibited DIF between the two modes. The overall rate of acceptance and preference for the touch-screen mode were quite high.

In order to develop a computerized version of the EORTC QLQ-PR25 for use in this study, we completed a required user’s agreement that permits us to use the questionnaire or for any change such as computerizing the questionnaire, and be held responsible for the quality of the measurement [33]. Permission was granted, per its policy, to allow us to use and migrate the paper form of the EORTC QLQ-PR25 to a tablet format for research purpose. Moreover, the U.S. Food and Drug Administration (FDA) released a PRO guidance in 2009, which suggested that a small randomized study is needed to provide evidence to confirm the new instrument’s adequacy for any change of instrument such as changing an instrument from paper to electronic format [34].

In our randomized cross-over designs, subjects were randomized into one of the two following sequences: paper then touch-screen or touch-screen then paper. Therefore, each subject served as his own control. Cross-over trials were conducted within participant comparisons, whereas parallel designs were conducted between participant comparisons. The influence of confounding covariates and the majority of between-patient variation could be eliminated using a cross-over design [34,35]. In this study, our results from mixed model analysis showed that no mode-by-order interaction effect was present; thus, the carry-over effect did not exist. Moreover, when we refitted the main effect by interaction term removal, the order effect did not exist. These results showed that the cross-over randomized design in our study is methodologically appropriate. Fewer patients may be required in the cross-over design to attain the same level of statistical power and precision. Moreover, this design permitted opportunities of head-to-head trials, and subjects receiving multiple treatments can express preferences for or against particular treatments [35,36].

In this study, we used intraclass correlation coefficients (ICCs), i.e., Pearson correlation coefficients when there were only two evaluations within a subject, to estimate the between-mode (touch-screen to paper; paper to touch-screen) test-retest reliability (0.40-0.84 for each item, 0.45-0.78 for each domain, and 0.80 for total score), which was consistently lower than the within-mode (paper-paper) test-retest reliability (0.61-0.93 for each item and 0.85 for total score) of the EORTC QLQ-PR25 questionnaire in another study [37]. The between-mode test-retest results may reflect the limitations of the original instrument rather than those of the data collection mode.

The equivalence testing that was used in reliability analysis was performed by two one-sided *t*-tests, which tested paired differences to be equal [38]. All the results from equivalence tests were significant indicating good equivalence, excluding Sexual functioning domain. It should be noted that of the six Sexual activity and functioning items in the EORTC QLQ-PR25, four items (SX52–SX55) are conditional and apply only to sexually active respondents. In this study, approximately half of the patients reported not being sexually active and did not respond to those 4 items, resulting in fewer responses to those conditional items in this domain. Moreover, quite a few responses were missing or showed variance in this domain due to the participants’ embarrassment or reluctance to share details about their private sexual life in this study population [39,40]. The above reasons may have resulted in less precise measurement in the Sexual functioning domain.

Moreover, DIF analysis was used to examine the difference in the difficulty papameters between two modes of administration (paper-and-pencil vs. touch-screen). Application of DIF analysis in this study allowed a thorough evaluation of measurement equivalence. We first estimated the difficulty calibration of each item for each mode separately using the rating scale model to avoid correlation problem. Then, DIF analysis was applied to assess the equivalence of item difficulties between these two modes. Although the problem of correlated data potentially existed, as the same patients in these two groups responded to the same survey twice due to the cross-over design, we mainly performed a comparison of the difficulty calibration measures, which is not so sensitive to the correlated effects of the cross-over design. For example, when performing the parallel comparison between two groups, the correlated data problem was mitigated to some extent due to the different sequence of administration, i.e., half the patients completed the questionnaire using the touch-screen mode first, followed by the paper mode, and the other half completed the paper mode first, followed by the touch-screen mode. In addition, the influence of confounding covariates and much of the between-patient variation could be eliminated using a cross-over design, which could also increase the efficiency for estimating and testing [35,41]. DIF also benefits from this type of design.

Furthermore, there are two possible reasons to explain the inconsistency between DIF analysis and equivalence/agreement analysis. First, these two approaches are conceptually different. DIF was used to test the scale measurement properties for each individual item between the two modes, while equivalence analysis was used to show the mean score difference of patients between two modes. Second, equivalence test as applied in this study began with a null hypothesis that the two mean values were not equivalent, and attempted to demonstrate that they were equivalent within a practical, preset limit. This test is conceptually opposite to the independent *t*-test in the DIF analysis and would lead to the result that the Sexual functioning items were more limited in equivalence/agreement, but the DIF test failed to identify this.

In this study, touch-screen administration required approximately 30% more time to complete than the paper questionnaire (20.5 min vs. 14.7 min). We speculate that the difference in time to complete the questionnaire may be because the touch-screen/paper group was given the touch-screen version questionnaire first, and most respondents (82%) had no experience using a computer. Furthermore, the patients may have spent more time because this was their first exposure to the EORTC QLQ-PR25 questionnaire; thus, the amount of time taken for the touch-screen mode was longer than that for the paper mode in the touch-screen/paper group (20.5 min vs 14.7 min) (*p* < 0.001). However, we found that when the touch-screen mode was performed in the second assessment, the time it took to complete the questionnaire decreased significantly (*p* = 0.002) (Table 1). We anticipate that the time to complete the questionnaire via touchscreen will decrease over time as patients get used to the system. In addition, the results can be analyzed in real-time to facilitate clinical diagnosis and improve patient-physician relationships. A previous study reported that the transfer of assessment data to a computer may differentially influence the responses of older patients [42]. Previous studies of patients aged from 48.1 years to 65.0 years showed the touch-screen mode was a reliable and user-friendly method of assessing quality of life [43-46]. Our results showed that 97% of the patients reported that the touch-screen version was user-friendly and approximately 67% reported preferring the touch-screen version to the paper-and-pencil version despite the older average age and lack of previous experience using a computer in our study population. Moreover, most patients (92%) reported that the touch-screen was easy to use, which was similar to the results reported by Pouwer et al. [43], which showed that the touch-screen questionnaire was easier for patients to complete even if they had rarely or never used a computer.

Patients often expect the physician to know the results of a questionnaire shortly after they have completed it; however, PROs assessed using the paper-and-pencil mode cannot be transferred to the physician’s clinic in real-time. Various studies have confirmed that incorporating routine standardized HRQOL assessments in clinical oncology practice can facilitate the communication and discussion of HRQOL issues and can increase physicians’ awareness of their patients’ quality of life. Computer measurements are well accepted by patients who generally consider ePROs to be useful tools with which to inform their doctor about their problems [47-49].

A limitation of this study was that the washout period between the two modes of administration was only 120 minutes, which might not have been sufficiently long to completely eliminate the carry-over effects. It is possible that some residual memory or carry-over effects from the first administration were still present when the patients were asked the same set of questions during the second administration. As a result, the level of agreement between the two administrations is possibly inflated [35,41]. A longer interval, however, may require patients to spend too much time waiting, which might therefore discourage them from completing the second administration. Asking patients to come back on another day would likely greatly reduce the subjects’ willingness to participate. Most importantly, because of the longer waiting time, the patients’ condition may change and result in different answers, thereby affecting the consistency of the responses. Therefore, after careful consideration, we chose an interval of 120 minutes. Moreover, once patients had finished the first questionnaire, they were taken to the education room to watch health education videos which helped to “dilute” the memory of the first administration. Thus, we believe that the memory effect was likely lessened due to the 120-minute interval between tests and the use of health education videos.

## Conclusions

Our results showed that the touch-screen mode of the Taiwan Chinese version of the EORTC QLQ-PR25 had demonstrated good reliability and was well accepted by most prostate cancer patients in Taiwan, suggesting its potential use as an alternative to the paper-and-pencil mode for measurement of PROs.

## Abbreviations

EORTC: European Organization for Research and Treatment of Cancer; EORTC QLQ-PR25: EORTC quality of life questionnaire prostate-specific 25-item; HRQOL: Health-related quality of life; DIF: Differential item functioning; PROs: Patient-reported outcomes; ePROs: Electronic patient-reported outcomes; US: Urinary symptoms; BS: Bowel symptoms; TS: Hormonal treatment-related symptoms; SX: Sexual activity and function; ICC: Intraclass correlations; MCID: Minimum clinically important difference; RSM: Rating scale model.

## Competing interests

All authors declare that they have no competing interests.

## Authors’ contributions

YJ Chang and CL Peng performed the statistical analyses and drafted the manuscript. WM Liang and CH Chang designed the study, wrote the protocol, and revised the manuscript. HC Wu was the coordinator of this research and conducted the field work. HC Lin, JY Wang, and TC Li participated in the design of the study, wrote the protocol, and supervised the execution of the study. YC Yeh was responsible for data collection and interpretation. All authors contributed to and approved the final manuscript.
